# Atlanto-axial rotary instability (Fielding type 1): characteristic clinical and radiological findings, and treatment outcomes following alignment, fusion, and stabilization

**DOI:** 10.1007/s10143-020-01345-9

**Published:** 2020-07-04

**Authors:** Fraser C. Henderson, Robert Rosenbaum, Malini Narayanan, Myles Koby, Kelly Tuchman, Peter C. Rowe, Clair Francomano

**Affiliations:** 1 Department of Neurosurgery, University of Maryland Capital Region Health Prince George’s Hospital Center, Cheverly, MD USA; 2 Doctors Community Hospital, Lanham, MD USA; 3 Metropolitan Neurosurgery Group LLC, Silver Spring, MD USA; 4 Department of Neurosurgery, Walter Reed-Bethesda National Military Medical Center, Bethesda, MD USA; 5 Department of Pediatrics, Johns Hopkins University, Baltimore, MD USA; 6 Medical and Molecular Genetics, Indiana University Health Physicians, Indianapolis, IN USA

**Keywords:** Atlanto-axial, Rotary subluxation, Dynamic imaging, Alar ligament, Syncope, C1-C2 fusion

## Abstract

Atlanto-axial instability (AAI) is common in the connective tissue disorders, such as rheumatoid arthritis, and increasingly recognized in the heritable disorders of Stickler, Loeys-Dietz, Marfan, Morquio, and Ehlers-Danlos (EDS) syndromes, where it typically presents as a rotary subluxation due to incompetence of the alar ligament. This retrospective, IRB-approved study examines 20 subjects with Fielding type 1 rotary subluxation, characterized by anterior subluxation of the facet on one side, with a *normal atlanto-dental interval*. Subjects diagnosed with a heritable connective tissue disorder, and AAI had failed non-operative treatment and presented with severe headache, neck pain, and characteristic neurological findings. Subjects underwent a modified Goel-Harms posterior C1-C2 screw fixation and fusion without complication. At 15 months, two subjects underwent reoperation following a fall (one) and occipito-atlantal instability (one). Patients reported improvement in the frequency or severity of neck pain (*P* < 0.001), numbness in the hands and lower extremities (*P* = 0.001), headaches, pre-syncope, and lightheadedness (all *P* < 0.01), vertigo and arm weakness (both *P* = 0.01), and syncope, nausea, joint pain, and exercise tolerance (all *P* < 0.05). The diagnosis of Fielding type 1 AAI requires directed investigation with dynamic imaging. Alignment and stabilization is associated with improvement of pain, syncopal and near-syncopal episodes, sensorimotor function, and exercise tolerance.

## Introduction

Atlanto-axial instability (AAI) results most commonly from trauma, due to injury of the transverse odontoid ligament, less commonly from congenital conditions such as os odontoideum or Klippel-Feil syndrome, and least commonly neoplasm or infection. The last few decades have witnessed increased recognition of AAI in the connective tissue disorders, including inflammatory disorders (such as rheumatoid arthritis, systemic lupus erythematosus, Stihl disease, ankylosing spondylitis) and heritable disorders, such as Ehlers-Danlos (EDS), Stickler, Loeys-Dietz, Marfan, and Morquio syndromes (Mucopolysacharridosis IV) [7]; spondyloepiphyseal dysplasia; and congenital hemifacial microsomia conditions, such as Goldenhar syndrome. AAI can also occur with minimal or no trauma in children as a result of ligament laxity [82]. And developmental conditions, such as atlanto-occipital segmentation failure—the HOX-D3 homeotic transformation—are also recognized as predisposing to AAI.

The atlanto-axial joint, the most mobile in the body, is stabilized primarily by ligaments [10]. Therefore, AAI is not uncommon in conditions of *lax ligaments*. Down syndrome has an 11 to 17% incidence of AAI [21, 29, 34, 66, 77]. Of Halko’s 4 subjects with vascular EDS, three had AAI [38]. One-third of children with ligamentous laxity due to congenital spondyloepiphyseal dysplasia suffered from AAI with cervical myelopathy [70]. Healey reported on eight subjects with Goldenhar syndrome, of which three had AAI [40]. The inflammatory connective tissue disorders are no exception: prior to the development of effective disease-modifying pharmacotherapies, 88% of rheumatoid arthritis patients exhibited radiographic evidence of C1-C2 involvement, 49% were symptomatic, 20% were myelopathic, and 10% succumbed to death [23, 68].

While others have reported open reduction of fixed subluxation in children with neck deformity but no significant functional or neurological symptoms [33], this study addresses Fielding type 1 rotary subluxations (AAI) in patients with substantial functional and neurological symptoms, in the context of hereditable connective tissue disorders. This cohort is comprised primarily of Ehlers-Danlos syndrome, hypermobility type (designated as h-EDS), in whom the diagnosis of AAI requires dynamic imaging, such as rotational CT of C1-2 to show pathological angular displacement, or lateral neck tilting to demonstrate lateral displacement [63].

AAI Fielding type 1 subluxations are characterized by anterior displacement of the facet primarily on one side, with a *normal atlanto-dental interval.* This form of AAI, found predominantly in the hereditary connective tissue disorder populations, is due to alar ligament incompetence, as opposed to the transverse ligament failure in rheumatoid arthritis and Down syndrome.

Lightheadedness was present in every patient, with syncope or pre-syncope reported by 75%. Measurement of pre-operative symptoms before and after stabilization of the C1-C2 motion segment supports the observation that the somatic and autonomic nervous system is materially and adversely effected by mechanical deformation of the neuraxis and instability at the C1-C2 level, and that there are substantial and salutary benefits to neurological function with the elimination of this deformity and instability.

## Materials and methods

### Subject enrollment

A cohort of 20 subjects diagnosed with EDS, or in one case, unspecified hereditary connective tissue disorders (HCTD), was enrolled retrospectively in an IRB approved study **(**Greater Baltimore Medical Center). All subjects had undergone C1-C2 reduction, fusion, and stabilization for atlanto-axial instability between 2017 and 2018 at a single institution by the authors (FCH, RR). The subjects were taken consecutively, with the exception that three patients (who did not respond to the lengthy questionnaires) were replaced with three subjects next on the list. The subjects were composed of 16 females and 4 males from 11 different states, age 18–54 years, average age 34 years at time of surgery.

### Evaluation

Most of the patients had been evaluated by the geneticist (CF). Pain was assessed by the visual analog scale for pain (0–10/10) before and after surgery (range 12–24 months). Neurological symptoms were assessed by a comprehensive standardized questionnaire. The neurologic exams were performed by the neurosurgeons (FCH, RR, MN).

Participants were contacted by a third party 12–24 months following surgery, and asked to recall the frequency of symptoms in the month before surgery and in the month before post-operative questionnaire completion, and these were cross-checked with the symptom pre-operative questionnaire for accuracy. The frequency of symptoms was reported using a 5-point scale (never, 1–3 times/month, once weekly, multiple times weekly, and daily). The severity of symptoms was ranked on a 4-point scale (no symptoms, mild, moderate, severe).

Respondents also completed a Karnofsky Performance score and the Short Form Health Survey (SF-36) physical functioning subscale (which assess difficulty with activities such as running, lifting, bending, stooping, walking several hundred yards to a mile and climbing several flights of stairs). Subjects recorded their ability to return to work. At follow-up, participants also completed a Global Clinical Impression of Change score, which asked them to grade the changes in activity, symptoms, and quality of life since the C1-C2 surgery using a 7-point scale (no change or condition has gotten worse, almost the same, hardly any change at all, a little better but no noticeable change, somewhat better but the change has not made any real difference, moderately better and a slight but noticeable change, better, and a definite improvement that has made a real and worthwhile difference, or a great deal better and a considerable difference that has made all the difference).

**Fig. 1:**
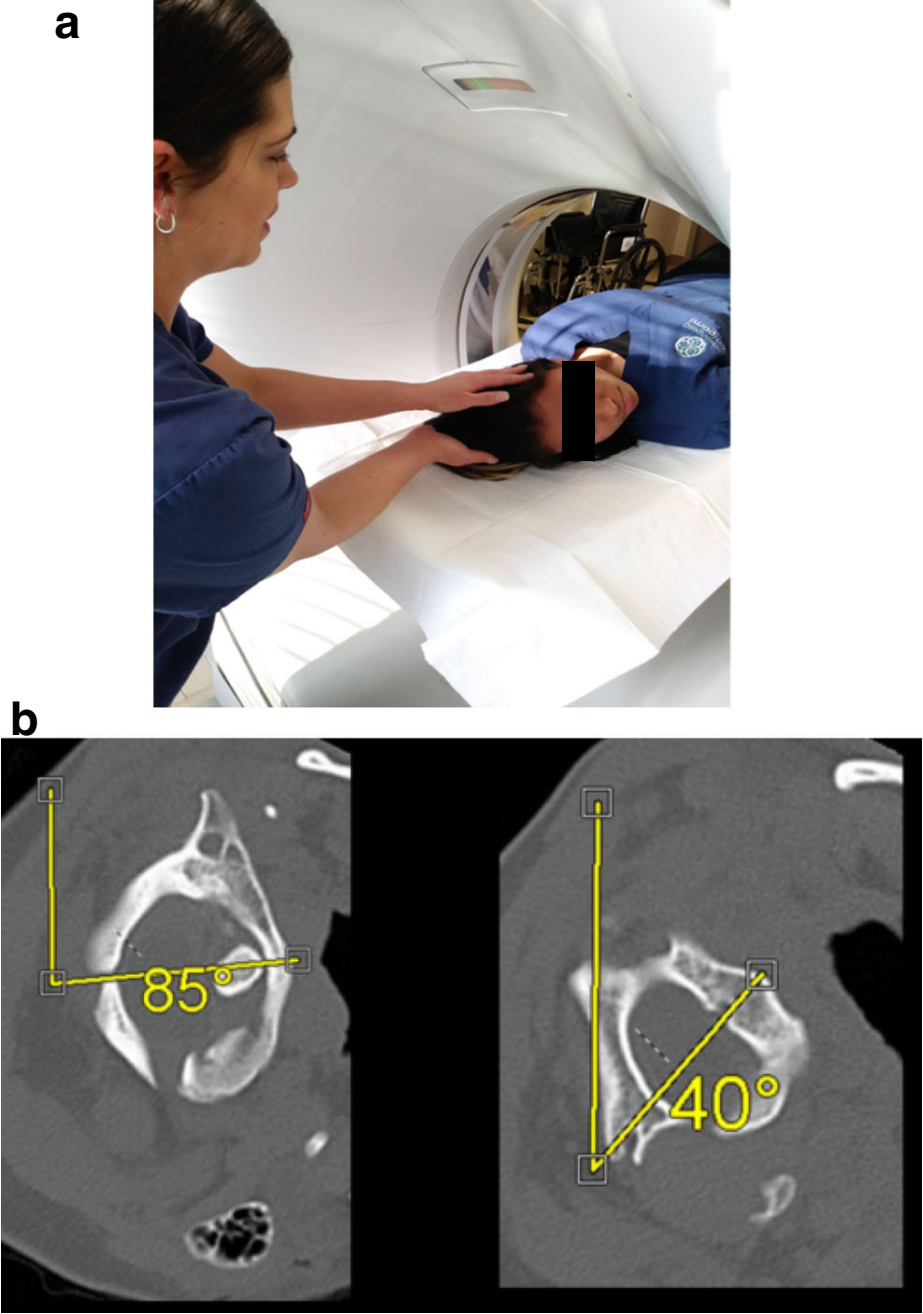
**a** To assess for alar ligament incompetence and possible subluxation between C1 and C2, a dynamic CT of the upper cervical spine is performed. A CT scan is performed with the patient lying supine, with the head rotated fully to the left, then rescanned with the head rotated fully to the right, as shown in **b**. **b** Axial CT view through C1 and C2, showing the angle deviation from the neutral position (12 o’clock position). The angle subtended by C1 minus the angle subtended by C2 is the angle between C1 and C2. In this case, the angle subtended between C1and C2 is 85° minus 40° equals 45°, which exceeds the pathological threshold of 42°

**Fig. 2:**
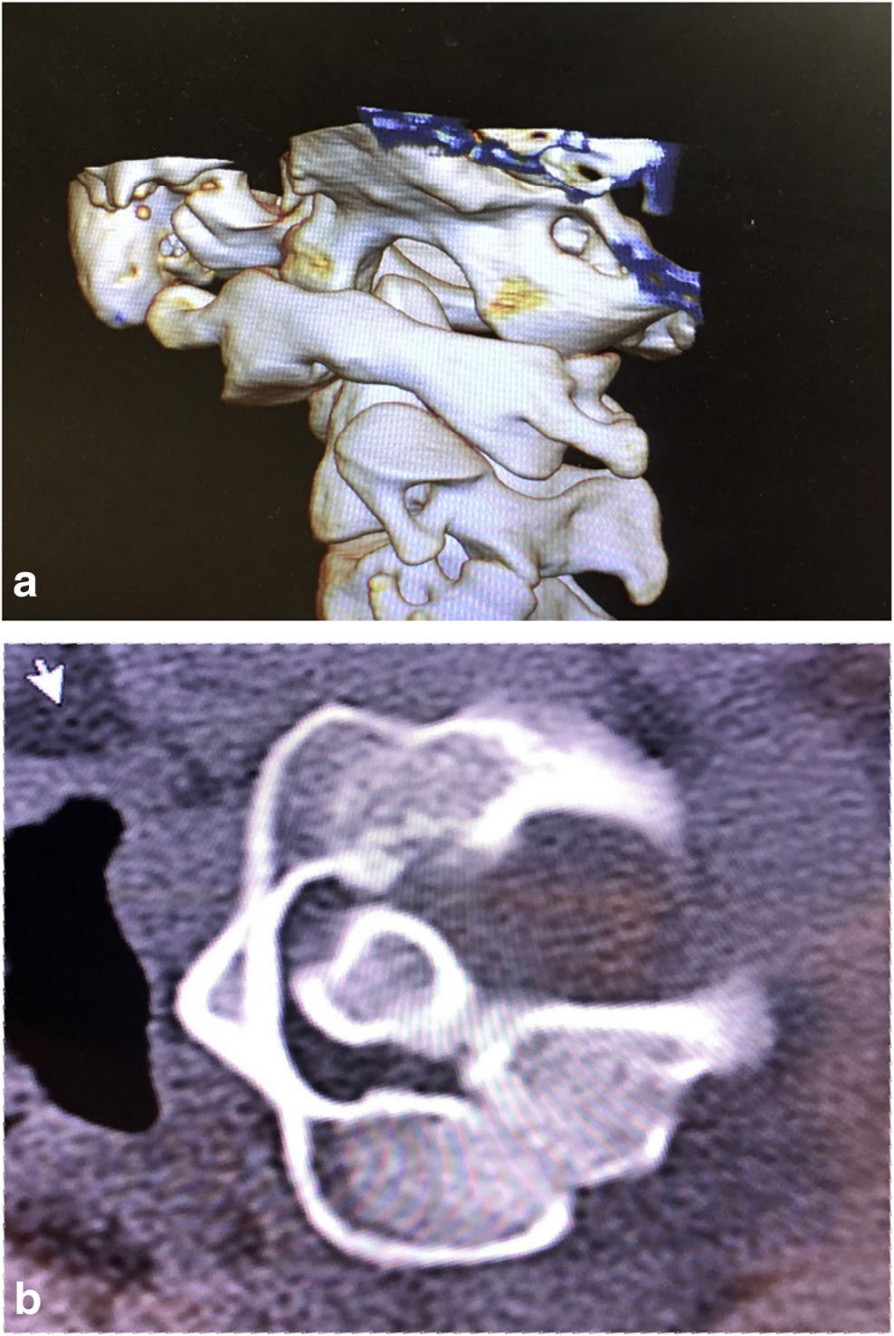
**a** A 3D CT reconstruction of the cervical spine rotated to the left shows that the left superior facet of C2 is more than 90% exposed, and, therefore, defined as subluxed. **b** Axial CT of C1 and C2 showing neck rotation to the right. Note the Fielding type 1 subluxation, with anterior subluxation of the left C1 facet (anterior to the left C2 facet), while the atlanto-dental interval—between the anterior tubercle and the odontoid—remains normal

Pre- and post-operative radiological measurements were made by the neuroradiologist (MK). Subjects underwent pre-operative computerized tomography (CT) of the cervical spine, with the neck maximally rotated (usually 75 to 90°) to the left and to the right (Fig. 1). The axial views of C1 and C2 upon full-neck rotation were measured from the 12 o’clock position; the angle of rotation of C2 is subtracted from the angle of rotation of C1. Angular displacement between C1 and C2 of > 41° was considered pathological. When 3D CT reconstruction was available, the percentage C1-C2 facet subluxation was assessed (Fig. 2). Post-operative CT was performed at 3–16 months to assess fusion and alignment.

### Statistical analysis

For comparisons of pre- and post-operative data for each participant, we used paired *t* tests for normally distributed data, and the Wilcoxon paired ranks test for paired ordinal data or for continuous variables that were not normally distributed. Statistical analyses were conducted using IBM Statistics SPSS version 25 (IBM Statistics, New York), and illustrations were prepared using GraphPad Prism version 8.3.0 for Windows (GraphPad Software, La Jolla, CA, USA, www.graphpad.com). *P* values reported as < 0.05 were in the 0.01 < *P* < 0.05 range, and *P* values reported as < 0.01 were in the 0.001 < *P* < 0.01 range.

### Inclusion criteria for C1-C2 fusion surgery

**Fig. 3:**
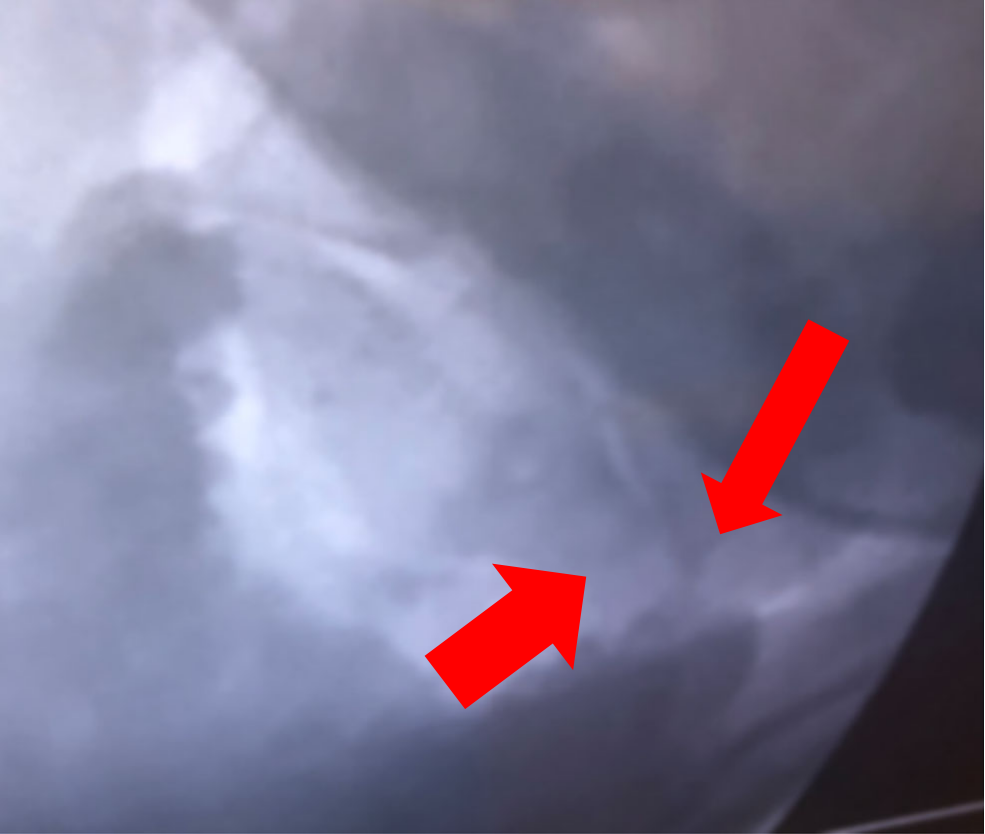
Open mouth antero-posterior view of digital dynamic imaging of the cervical spine shows a pathological translation of 5 mm to the right (see arrows), which exceeds the normal reference range (< 2 mm)

All subjects met the following criteria:

i. Formal genetics evaluation and diagnosis with a hereditary connective tissue disorder.
ii. Severe headache and/or neck pain for greater than 6 months.
iii. Symptoms compatible with atlanto-axial instability [5, 69].
iv. Congruent neurological deficits.
v. Radiological findings—an angle subtended by C1-C2 greater than 41° (Fig. 1b), and/or C1-C2 facet overlap of less than 10% (Fig. 2a and b). In some cases, radiological findings were augmented by fluoroscopic demonstration of pathological translation on lateral tilt > 3.5 mm on open mouth views [18, 26]. (Fig. 3)
vi. Failed conservative treatment, including a reasonable trial of physical therapy, activity modification, pain medications, neck brace, and other modalities.

### Conservative treatment

Conservative therapy was recommended to every subject, and subjects were told that surgery was to be undertaken only if non-operative therapy failed. Our goal was to optimize alignment with brace, to strengthen the neck, and to avoid injurious activities. Most patients were fitted with a neck brace (usually the Aspen Vista® Cervical Collar), directed to wear it for 23 h a day for 4 weeks, and given detailed instructions on the performance of *isometric exercises* to strengthen the neck. They were instructed to maintain good neck posture at all times and to avoid neck bending, prolonged sitting, driving, and any activities involving contact sports, deceleration, or strenuous activities which could harm the neck. Physical therapy was ordered, with the caveat that range of motion exercises and manipulation should be avoided. Several subjects with very severe symptoms had already undergone a protracted period of non-operative therapy and were not compelled to repeat the conservative therapy regimen. In several cases, the neck brace was discontinued because of pressure against the mandible, causing severe pain in the temporomandibular joint.

### Operative technique

**Fig. 4:**
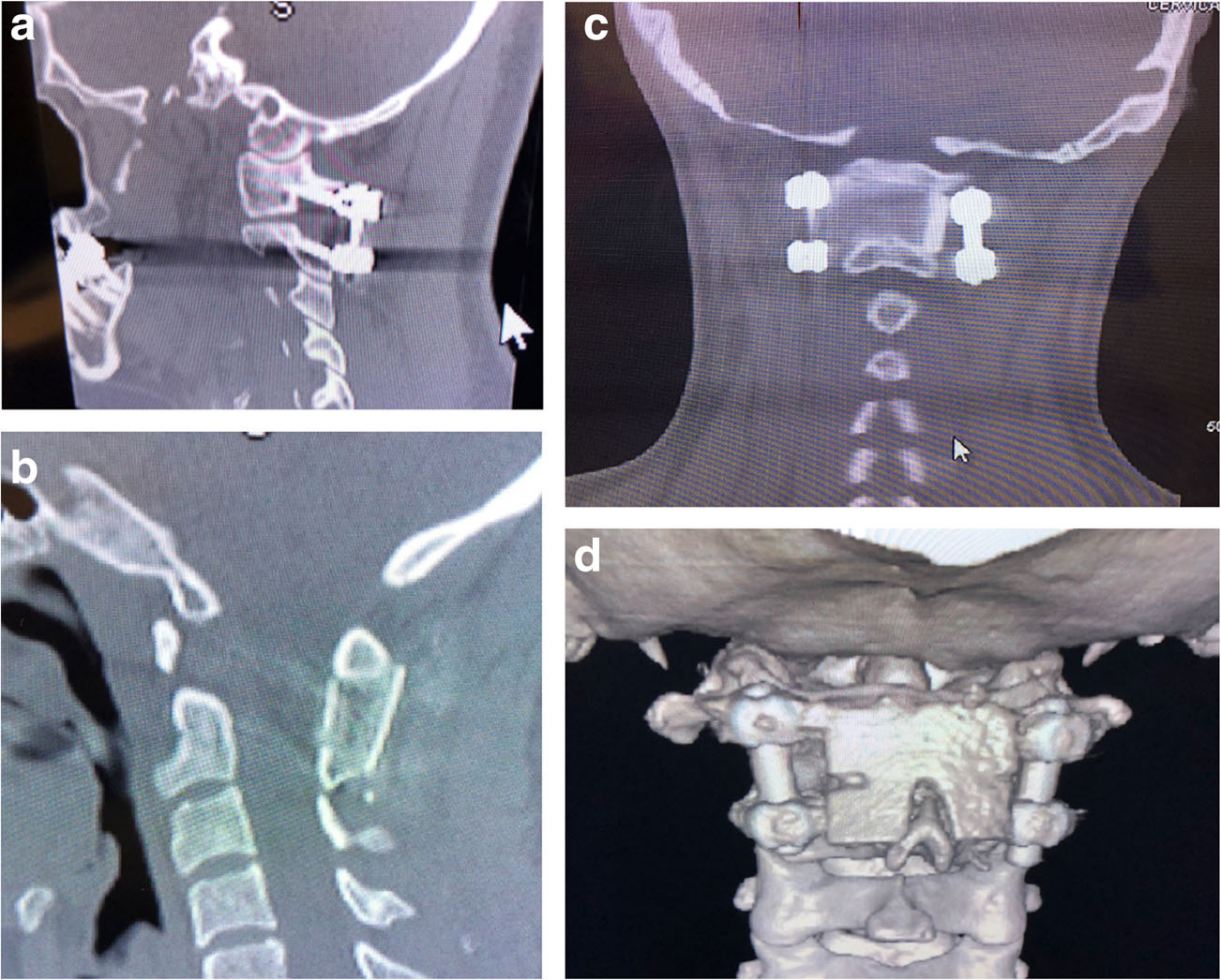
**a** Sagittal reconstruction of the cervical spine CT showing placement of the C1-C2 screws and connecting rods (Goel-Harms technique of C1-C2 stabilization). **b** CT mid-sagittal reconstruction showing the C1-C2 fusion. Note the allograft encompasses the posterior ring of C1 and tightly abuts the spinous process and lamina of C2. **c** Coronal view of CT showing graft placement between the posterior ring of C1 above and the spinous process and lamina of C2 below, and between the screws at C1-C2 bilaterally. **d** A 3D CT reconstruction of the upper cervical spine showing the C1-C2 fusion and stabilization from a posterior perspective.

The surgical technique, described by Goel and Harms, was modified [3, 30]. The C1 screw entry points, measured on the pre-operative CT scan from the midpoint, were drilled and tapped toward the anterior tubercle of C1 as seen on sagittal view, angling approximately 8° medially. At the C2 level, screws were angled medially 30°, and, superiorly, parallel to the cortex of the superior facet of C2. Twenty-four-millimeter smooth shank screws were placed at C1, and fully threaded 24-mm screws at C2 (Solstice®, LifeSpine Inc., Huntley, IL), except in those cases where the large size of the vertebral artery foramen, or small pedicle, militated for a shorter screw. An intraoperative fluoroscopic-CT scan ensured correct screw placement. Bone marrow, harvested by aspiration from the iliac crest, was infused into a saline-soaked, tri-cortical, iliac crest allograft; the allograft was contoured precisely to fit the decorticated, posterior ring of C1, and the lamina and spinous process of C2 **(**Fig. 4a–d).

### Post-operative management

Patients were encouraged to stand at the bedside on the evening of the surgery, and to ambulate on post-operative day 1. The wound drain was removed on post-operative day 2 or 3. Intravenous opiate (dilaudid or morphine), administered by patient-controlled administration (PCA), was weaned off on post-op day 2; oral opiates and Ketorolac® 10 mg (every 6 h) were given on the third day. Patients wore a neck brace for 1 month after surgery, and then started on physical therapy for posture, core strengthening, cardiovascular fitness, and isometric exercises of the neck. Subjects were asked to maintain strict neck posture. Range of motion exercises was discouraged in this cohort to avoid potential ligamentous injury to the lower cervical levels. Follow-up was conducted at 2 weeks, and at 3 months, at which time a CT of the cervical spine was performed.

### Statement of human and animal rights

All procedures performed in studies involving human participants were carried out in accordance with the ethical standards of the institutional and/or national research committee in the USA, and with the 1964 Helsinki declaration and its later amendments or comparable ethical standards. Informed consent was obtained from all individual patients and participants included in the study.

## Results

### Study subjects

Included in the study were 20 subjects (4 males, 16 females), ages 18–54 years, average age 34 years at time of surgery from 11 different states in North America. The subjects underwent atlanto-axial reduction, fusion, and stabilization between 2017 and 2018 by the senior authors (FH, RR).

### Neurological findings

Four symptoms commonly occurred in this cohort: headaches in the distribution of the occipital nerves (unilateral or bilateral, exacerbated by looking up or down, and driving upon uneven surfaces): frequent syncopal or pre-syncopal episodes on a daily or weekly basis (the subjects reported learning to anticipate a syncopal episode, and undertaking immediate avoidance strategies), visual changes (including teichopsia, decreased peripheral, or “tunnel” vision; most subjects reported walking into door frames, benches, tables, or people), many reported “a brownout” or extreme blurring of vision, and finally, intermittent dysesthesias of the extremities. All subjects reported poor concentration and memory, nausea, and tinnitus.

Three physical findings were commonly present. First, moderate or severe tenderness to pressure over C1, which often provoked nausea. Second, hypoesthesia to pinprick over most of the cervical, thoracic, lumbar, and sacral dermatomes, in which the subject reported that the pin was pointed, but not painful. Proprioception and vibratory sensation were rarely impaired. Third, hyperreflexia, especially of the patellar tendons (12/20), except in those subjects with peripheral neuropathy due to B12 deficiency (8/20) who demonstrated hyporeflexia. Less common were weakness (9/20), dysdiadochokinesia (5/20), Romberg sign (6/20), and impairment of tandem gait (7/20).

### Radiological findings pre-operatively

The average angle between C1 and C2 on full-neck rotation to each side was 42° (Fig. 1a and b). Significant loss of facet overlap (that is, less than 20% facet overlap) was demonstrated in all subjects (less than 10% in four, less than 5% overlap in two) (Fig. 2). Several patients also underwent open mouth, antero-posterior views showing excessive translation on lateral tilt (exceeding 3.5 mm to left and/or right) (Fig. 3).

### The Karnofsky Performance score (KPS)

**Fig. 5:**
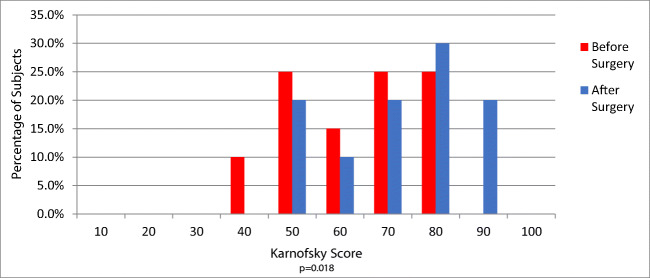
Histograms comparing pre-operative and post-operative Karnofsky Performance scores shows a statistically significant improvement of performance (*P* = 0.02) after the C1-C2 fusion stabilization; most patients were able to return to work or school

Improvement in performance reached statistical significance (*P* = 0.022, with 50% of subjects returning to work (Fig. 5).

### Improvement in the Short Form Health Survey

Patients reported statistically significant improvement in activities such as running, lifting (*P* = 0.01), bending, stooping (*P* < 0.05), and walking several hundred yards to a mile (*P* = 0.02) (Table 1). The total mean (SD) SF-36 physical function raw score (which can range from 10 to a maximum of 30) improved from 16.6 (5.1) pre-surgery to 20.4 (5.3) post-surgery (*P* = 0.02).

**Table 1:** Comparison of SF-36 PF subscale pre- vs. post-surgery

Limitation measure	% with limitation pre-op	% with limitation post-op	% with improvement post-op*	% with worsening post-op^†^	% with onset post-op	*P* value^‡^
Vigorous activities (running, lifting, etc.)	100%	95% (19/20)	45% (9/20)	5% (1/20)	0	0.01
Moderate activities (vacuuming, bowling, etc.)	95% (19/20)	80% (16/20)	52.7% (10/19)	5.3% (1/19)	5% (1/20)	0.14
Lifting or carrying groceries	85% (17/20)	65% (13/20)	58.8% (10/17)	11.8% (2/17)	5% (1/20)	0.11
Climbing several flights of stairs	90% (18/20)	83.3% (17/20)	38.9% (7/18)	5.6% (1/18)	5% (1/20)	*0.10*
Climbing one flight of stairs	65% (13/20)	60% (12/20)	45% (9/20)	0	30% (3/20)	0.17
Bending, kneeling, stooping	85% (17/20)	75% (15/20)	52.9% (9/17)	17.6% (3/17)	5% (1/20)	< 0.05
Walking more than a mile	90% (18/20)	75% (15/20)	47.1% (9/18)	5.6% (1/18)	5% (1/20)	*0.09*
Walking several hundred yards	75% (15/20)	50% (10/20)	66.7% (10/15)	6.7% (1/15)	5% (1/20)	0.02
Walking 100 yards	55% (11/20)	35% (7/20)	72.3% (8/11)	0	5% (1/20)	0.11
Bathing and dressing yourself	60% (12/20)	45% (9/20)	58.3% (7/12)	0	10% (2/20)	0.18

*Where present pre-op

^†^For those participants who had presence of symptom/problem prior to surgery

^‡^Wilcoxon signed-rank test

### Symptoms showing significant improvement after surgery

As displayed in Table 2, patients reported a statistically significant improvement in the frequency and severity of headaches (Figs. 8 and 9) and neck pain (Fig. 10), hand numbness, lower extremity numbness, arm weakness, syncope, and pre-syncope, and to a milder extent in joint pain, myalgias, nausea, anxiety, and depression. There was also improvement in the frequency of lightheadedness, vertigo, brain fog, and palpitations, and in the severity of exercise intolerance.

**Table 2:** Frequency and severity of symptoms demonstrating significant improvement (*n* = 20)

Symptom/problem	% pre-surgery	% post-surgery	% with improvement in frequency post-surgery*	% with worsening of frequency post-surgery*	Frequency *P* value^†^	% with onset post-surgery	% with improvement severity post-surgery*	% with worsening of severity post-surgery*	Severity *P* value^†^
Neck pain	100%	100%	50% (10/20)	0	< 0.01	0	75% (15/20)	0	< 0.001
Headaches	100%	100%	55% (11/20)	10% (2/20)	< 0.05	0	65% (13/20)	5% (1/20)	< 0.01
Occipital neuralgia	85% (17/20)	80% (16/20)	52.9% (9/17)	5.8% (1/17)	0.01	0	58.8% (10/17)	5.8% (1/17)	< 0.01
Muscle pain	100%	100%	35% (7/20)	0	< 0.05	0	40% (8/20)	10% (2/20)	< 0.05
Joint pain	95% (19/20)	95% (19/20)	26.3% (5/19)	0	< 0.05	0	31.6% (6/19)	0	< 0.05
Numbness hands	95% (19/20)	85% (17/20)	68.4% (13/19)	0	0.001	0	68.4% (13/19)	0	0.001
Numbness elsewhere (LE)	95% (19/20)	80% (16/20)	68.4% (13/19)	0	0.001	0	57.9% (11/19)	0	< 0.01
Weakness arms	75% (15/20)	60% (12/20)	60% (9/15)	13.3% (2/15)	0.01	0	53.3% (8/15)	13.3% (2/15)	< 0.05
Lightheadedness	100%	100%	55% (11/20)	0	< 0.01	0	40% (8/20)	10% (2/20)	0.07
Palpitations	80% (16/20)	75% (15/20)	37.5% (6/16)	6.3% (1/16)	< 0.05	0	43.8% (7/16)	12.5% (2/16)	0.07
Dizziness	100%	100%	60% (12/20)	15% (3/20)	< 0.05	0	50% (10/20)	15% (3/20)	0.07
Pre-syncope	90% (18/20)	55% (11/20)	61.1% (11/18)	11.1% (2/18)	< 0.01	0	61.1% (11/18)	11.1% (2/18)	< 0.05
Syncope	45% (9/20)	25% (5/20)	66.7% (6/9)	0	< 0.05	0	66.7% (6/9)	0	< 0.05
Brain fog	100%	100%	40% (8/20)	0	0.01	0	40% (8/20)	20% (4/20)	0.15
Tremors	60% (12/20)	60% (12/20)	50% (6/12)	0	0.05	5% (1/20)	58.3% (7/12)	8.3% (1/12)	0.10
Vertigo	75% (15/20)	65% (13/20)	60% (9/15)	0	0.01	5% (1/20)	53.3% (8/15)	13.3% (2/15)	0.05
Nausea	90% (18/20)	75% (15/20)	61.1% (11/18)	11.1% (2/18)	< 0.05	0	44.4% (8/18)	16.7% (3/18)	< 0.05
Exercise intolerance	85% (17/20)	80% (16/20)	52.9% (9/17)	5.9% (1/17)	0.06	0	64.7% (11/17)	11.8% (2/17)	< 0.05
Anxiety	90% (18/20)	85% (17/20)	55.6% (10/18)	11.1% (2/18)	< 0.05	0	44.4% (8/18)	11.1% (2/18)	< 0.05
Depression (EDS)	85% (17/20)	85% (17/20)	58.8% (10/17)	10% (2/20)	0.01	0	52.9% (9/17)	11.8% (2/17)	< 0.05

*For those subjects who had symptom/problem prior to surgery

^†^*P* values refer to the comparison of the pre-operative and post-operative scores provided by all 20 patients for the frequency (on a 5-point scale) and severity (on a 4-point scale) for each symptom (Wilcoxon signed-rank test), with values < 0.05 indicating significant improvement in that symptom following surgery

### Symptoms with no improvement

There was no significant improvement in fatigue, memory, balance, swallowing/choking, leg weakness, or sexual difficulties (Table 3).

**Table 3:** Frequency and severity of symptoms demonstrating no significant improvement (*n* = 20)

Symptom/problem	% pre-surgery	% post-surgery	% with improvement in frequency post-surgery*	% with worsening of frequency post-surgery*	Frequency *P* value^†^	% with onset post-surgery	% with improvement severity post-surgery*	% with worsening of severity post-surgery*	Severity *P* value^†^
Fatigue	100%	100%	35% (7/20)	10% (2/20)	*0.07*	0	55% (11/20)	15% (3/20)	0.10
Poor memory	90% (18/20)	95% (19/20)	44.4% (8/18)	0	0.23	5% (1/20)	27.8% (5/18)	16.7% (3/18)	1
Loss vision	75% (15/20)	60% (12/20)	46.7% (7/15)	13.3% (2/15)	*0.08*	0	40% (6/15)	20% (3/15)	0.17
Hands/feet cold/blue	70% (14/20)	70% (14/20)	35.7% (5/14)	7.1% (1/14)	*0.07*	0	21.4% (3/14)	7.1% (1/14)	0.33
Difficulty balancing	80% (16/20)	70% (14/20)	68.8% (11/16)	12.5% (2/16)	*0.10*	10% (2/20)	*68.8% (11/16)*	6.2% (1/16)	*0.06*
Difficulty walking	70% (14/20)	75% (15/20)	64.3% (9/14)	7.1% (1/14)	0.66	15% (3/20)	64.3% (9/14)	7.1% (1/14)	0.32
Leg weakness	70% (14/20)	75% (15/20)	57.2% (8/14)	14.3% (2/14)	0.63	10% (2/20)	57.2% (8/14)	7.1% (1/14)	0.32
Dysphagia	70% (14/20)	80% (16/20)	50% (7/14)	21.4% (3/14)	0.69	15% (3/20)	28.6% (4/14)	21.4% (3/14)	0.69
Choking	35% (7/20)	45% (9/20)	28.6% (2/7)	14.3% (1/7)	0.71	16.7% (3/18)	28.6% (2/7)	14.3% (1/7)	0.65
Hearing problems	70% (14/20)	65% (13/20)	35.7% (5/14)	28.6% (4/14)	0.63	10% (2/20)	28.6% (4/14)	21.4% (3/14)	0.81
Urinary frequency	70% (14/20)	75% (15/20)	7.1% (1/14)	21.4% (3/14)	0.27	5% (1/20)	14.3% (2/14)	14.3% (2/14)	0.67
Urinary incontinence	50% (10/20)	40% (8/20)	30% (3/10)	30% (3/10)	1	0	40% (4/10)	30% (3/10)	0.79
Sexual dysfunction	40% (8/20)	35% (7/20)	25% (2/8)	12.5% (1/8)	0.48	5% (1/20)	37.5% (3/8)	12.5% (1/8)	1

*For those subjects who had presence of symptom/problem prior to surgery

^†^*P* values refer to the comparison of the pre-operative and post-operative scores provided by all 20 patients for the frequency (on a 5-point scale) and severity (on a 4-point scale) for each symptom (Wilcoxon signed-rank test)

### Patient’s Global Impression of Change

**Fig. 6:**
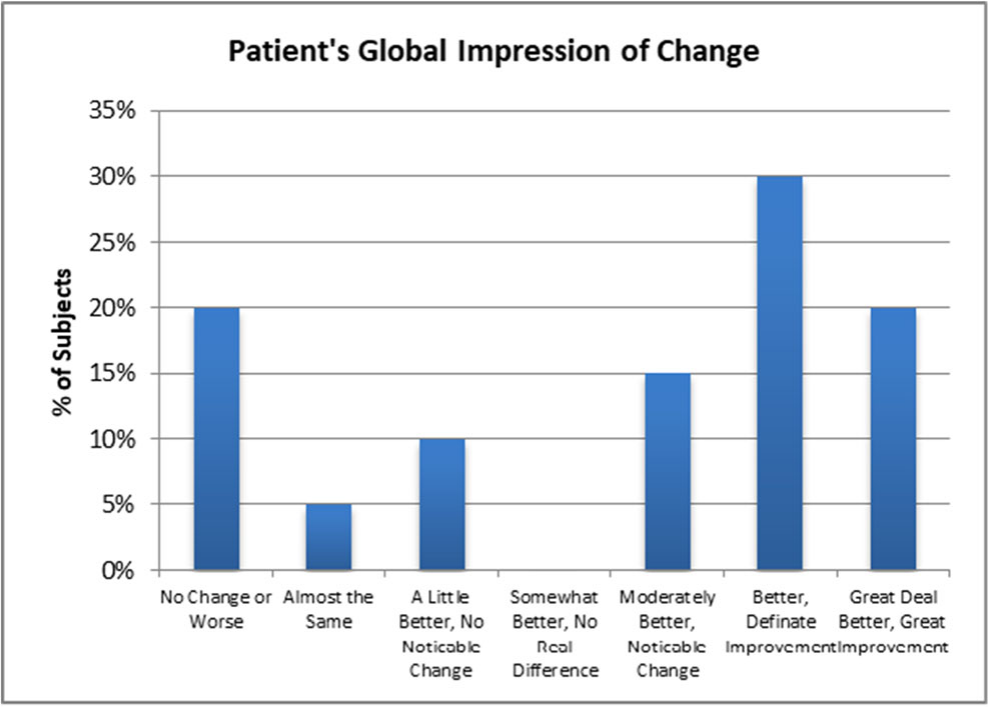
Patient’s Global Impression of Change; graph histograms show that 65% of subjects reported improvement, and 35% of subjects reported no significant improvement of overall symptoms. (The failure of many to improve was thought in part to be the result of the many comorbid conditions experienced by the subjects)

Sixty-five percent of subjects reported an improvement as measured by the Global Clinical Impression of Change (Fig. 6). Thirty-five percent of subjects reported no significant overall improvement. Global pain (total body, joint, spine, head, viscera) was also assessed with the visual analog scale (VAS): one reported worse pain, 7 were unchanged, the remaining 12 were improved. The overall pre-operative VAS of global pain was 7/10, and the post-operative global pain (VAS) was 6/10 (*P* = 0.001).

### Patient satisfaction surveys

**Fig. 7:**
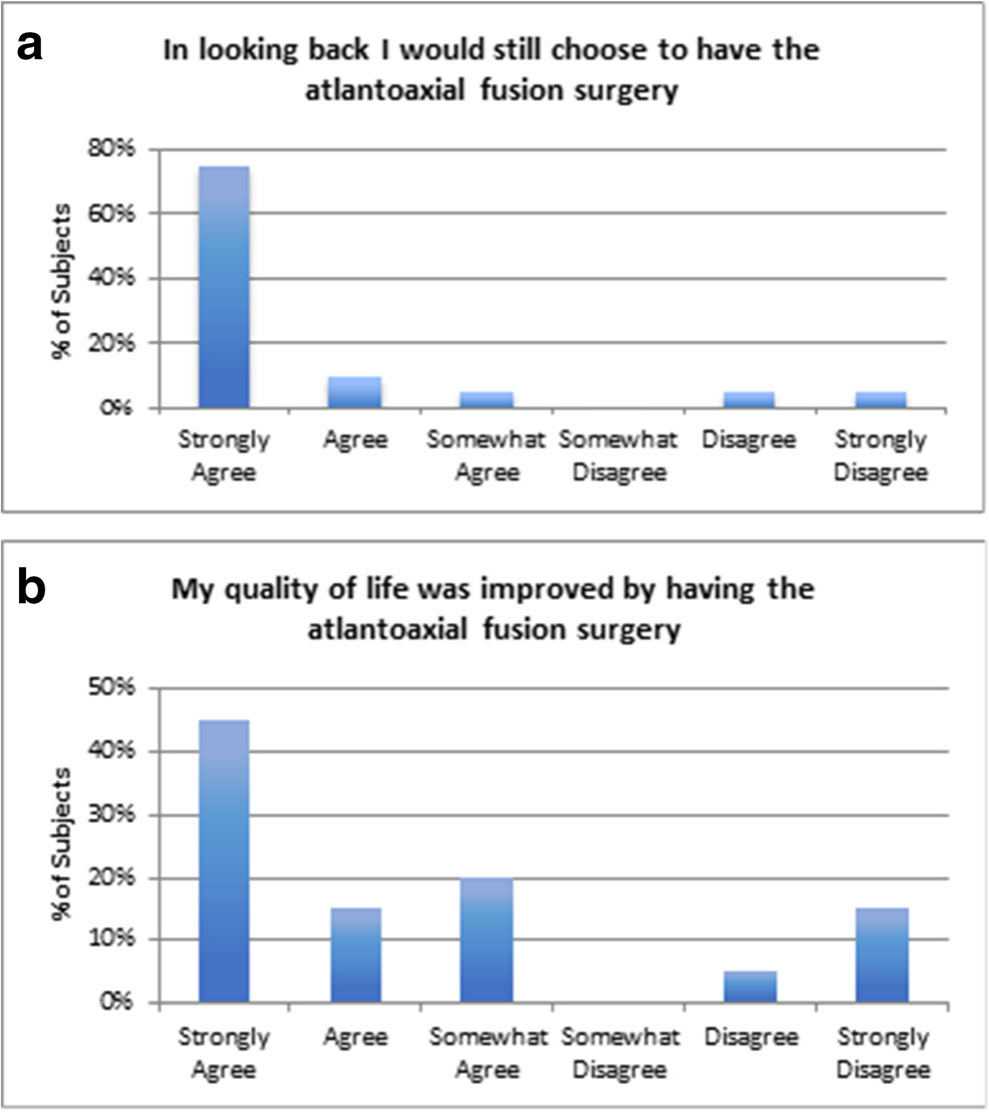
**a** Histogram showing patient assessment of surgery; in looking back, 90% of subjects would repeat the surgery given the same circumstances, and 10% (2 patients) would not repeat the surgery. **b** Histograms of patient assessment of quality of life, showing that 80% enjoyed an improved quality of life, whereas 20% did not

Overall, 90% of patients indicated they would repeat the surgery given the same circumstances, and 10% would not repeat the surgery, citing the presence of continued comorbidities (Fig. 7a). Four of the 20 subjects (20%) stated that the surgery did not help their overall quality of life (Fig. 7b).

### Post-operative fusion

Eighteen fusions (18/20) were confirmed by CT. One subject chose not to undergo post-op CT, but had no complaints. One subject experienced trauma and returned with failed fusion; a second subject returned with a broken screw, but evidence of good fusion (see below).

### Complications

There were no peri-operative complications within the first month. One subject fell at 1 year and required revision of the C1-C2 fusion for broken graft. A second subject showed evidence of fusion at 6 months, but at 1 year, she demonstrated a broken C2 screw and exhibited clinical and radiological findings of cranio-cervical instability; she later required extension of the fusion to include the occiput (occiput-C2 fusion).

In the year after surgery, one subject underwent ICP monitoring for intracranial hypertension, one received a lumbo-peritoneal shunt, one occipital nerve neurolysis, and two were being evaluated for further lower level cervical spine pathology.

Every subject (20/20) suffered from ongoing orthostatic intolerance and at least one other comorbidity; these included mast cell activation syndrome, peripheral neuropathy, Chiari I malformation, transverse sinus stenosis, trigeminal neuralgia, epilepsy, cervical myelopathy, lower spinal CSF leak, SI joint dysfunction, Tarlov cyst syndrome, spina bifida occulta, fibromyalgia, digestive disorders (need J-tube/feeding tubes), esophageal dysmotility, gastroparesis, eosinophilic esophagitis, and interstitial cystitis. One subject was diagnosed with Gilbert syndrome, one with labyrinthitis, one Von Willebrand disease, and one parotid tumor.

## Discussion

### Characteristic symptoms and signs of AAI

In this study, AAI most commonly presented with four symptoms: severe headaches, syncope or pre-syncope, dysesthesias, and tunnel vision. In addition, most subjects reported nausea, tinnitus, and cognition issues. These symptoms should prompt consideration of AAI, especially in a patient with hypermobility issues.

Three findings were common in AAI: first, C1-C2 tenderness; second, ubiquitous hypoesthesia to pinprick; third, hyperreflexia, with the caveat that subjects with peripheral neuropathy did not have hyperreflexia. Weakness, dysdiadochokinesia, and altered tandem gate were often present, and not dissimilar to those described in the literature for AAI [3, 16, 30, 32, 48].

### Radiologic diagnosis of AAI

Alar ligament incompetence underlays AAI in this cohort, consistent with the literature [38, 42, 50]. The subluxations are Fielding’s type 1, with anterior displacement (subluxation) of the facet on one side, and a normal atlanto-dental interval (ADI less than 3 mm) (Fig. 2a and b), reducible, and best-fused posteriorly [26, 88] (Fig. 4a–d).

Failure to diagnose AAI resides in the difficulty of recognizing *rotary instability* on standard x-ray, CT, and MRI images [57]. The use of rotational CT to diagnose rotary subluxation was established with cadaveric studies, which demonstrated a mean rotation between atlas and axis of 31.1°, increasing to 35° after contralateral rupture of the alar ligament [18]. In adults, there is vertebral artery obstruction at 40° rotation. For the purposes of this study, the diagnosis of AAI was based upon an angular displacement between C1 and C2 ≥ 41° as seen on CT axial views [18–20, 42, 44, 56, 74] (Figs. 1b and 2a and b).

Severe pain may limit the range of motion, in which case a rotation CT will be non-diagnostic; in these subjects, AAI can be alternatively demonstrated on lateral head tilt (Fig. 3). Lateral translation of more than 1.6–3 mm “offset” at the lateral margins of C1 on C2 is considered pathological [28, 46, 47, 84]. The authors have used a translation of ≥ 3.5 mm of the lateral mass of C1 upon C2, as seen on open mouth views during lateral neck tilt, as the pathological threshold in the demonstration of AAI [50] (Fig. 3).

Other techniques assess instability on the basis of the offset of the odontoid between the lateral masses; asymmetric distance between the lateral mass and dens is a common finding [28].

Loss of facet overlap (< 20%) is an indicator of rotatory subluxation. A 3D CT could also be helpful to demonstrate subluxation [27] (Fig. 2a). Cineroentgenography is helpful in diagnosing rotary subluxation [90].

Diagnosis of AAI may be made on the basis of compromise of the vertebral arteries, anomalous joints, and retro-odontoid pannus [32, 48, 51, 97]. Ligamentous disruption of the alar and odontoid ligaments is associated with increased signal intensity on high-resolution proton density–weighted MRI [58, 81].

### Indications for AAI surgery

In the general population, fusion is indicated when non-operative therapy fails to maintain normal correction and ensure stability, to maintain correction in the presence of central nervous system compromise—even if transient—and to maintain a normal C1-C2 relationship [26]. Before recommending surgery for AAI in the hereditary connective tissue population, the authors would add the requirements, with some exceptions, of severe neck or suboccipital pain (usually ≥ 7/10), appropriate neurological symptoms and findings, and congruent radiological findings, with the caveat that dynamic imaging is usually necessary to demonstrate ligament incompetence [42]. Moreover, atlanto-axial fusion should be considered the last option, only after the patient has undergone a reasonable trial of non-operative treatments.

### AAI surgery

The authors utilized the Goel-Harms technique, utilizing a C1 lateral mass screw, C2 pedicle screw fixation, and rods to stabilize the atlanto-axial joint (Fig. 4a–d). This technique is biomechanically reliable, precise, permissive of reduction, and adaptable to most anatomical variations. Moreover, this construct can be readily extended to the occiput or to the subaxial spine at a later date [1, 9, 14, 15, 31, 39, 95].

Surgery in this cohort of EDS subjects was more difficult for three reasons. First, the bone quality, though adequate to proceed with fusion, was generally osteopenic. Serum testing showed that many subjects were deficient in vitamin D, in which case they were prescribed large doses of vitamin D (®Ergocalciferol, 50,000 μ weekly). Subjects with osteoporosis were prescribed calcitonin nasal spray (1 puff daily) and calcium citrate. Second, the frequent comorbidities inherent to EDS required a more comprehensive medical management. Third, bone structures were smaller than average, requiring very accurate placement of screws. Thus, careful study of the CT scan and MRI is necessary prior to placing screws. The hereditary connective tissue disorders are associated with less robust bone anatomy, and a vertebral artery foramen closer to midline. Many fall in the category of patients in whom the pedicles of C2 are very narrow pose greater risk for neurovascular complications with pedicle screw placement [72]. It may be necessary to place a shorter pedicle screw on the side of the dominant vertebral artery.

The authors have identified common pitfalls related to fusion for AAI. First, a small C1 sagittal diameter may predispose to iatrogenic canal stenosis upon placement of the graft; this is obviated by concave sculpting on the underside of the graft. Second, a diminutive posterior ring of C1 has little cancellous bone and requires very precise fashioning of the bone graft to achieve fusion. Third, the C1 lateral mass screw should be angled toward the *lower* third of the anterior tubercle (as seen on sagittal view), to ensure that the screw does not transgress the atlanto-condylar joint. Fourth, segmentation failure between occiput and C1—the HOX-D3 homeotic transformation—requires fusion of the occiput to C2. Fifth, a Klippel-Feil anomaly at the C2-C3 level is associated with a small C2 pedicle, requiring instrumentation to include C3. Fifth, an overly lordotic C1-C2 segment results in a compensatory iatrogenic, subaxial kyphosis, swan neck deformity, or subaxial subluxation post-operatively; conversely, C1-C2 kyphosis results in subaxial hyperlordosis [64].

Anatomical reduction to restore optimal alignment of C1-C2 is necessary. Incomplete reduction risks injury to the vertebral arteries [60]. The 7% incidence of the posterior inferior cerebellar artery arising in the extra-dural location, and the 2.3% incidence of other anomalies, such as the pro-atlantal variants, should be kept in mind [85]. The *ponticulus posticus*, or Kimmerle anomaly, is common—19.3% in one series—and associated with a higher risk of VA injury [45]. Abnormal alignment of C1 and C2 may narrow the spinal canal and obstruct cerebrospinal fluid flow [65].

### Neurological outcomes after fusion stabilization

Neurological symptoms improved after the C1-C2 fusion stabilization. Improvement reached statistical significance in neck pain and headaches. The surprising improvement in joint and muscle pain is ascribed to improved neuromuscular control. There was statistically significant improvement in nausea, dizziness, lightheadedness, anxiety, bowel incontinence, and palpitations. The authors postulate that the nausea and vertigo in this population may sometimes result from intermittent compromise of the vertebral artery with transient ischemia of the peripheral labyrinth. Intermittent ischemia can result from abnormal C1-C2 rotation or alignment, occipital bone anomaly, hypertrophy of the atlanto-occipital membrane, tightness of the paravertebral muscles, or constriction by fibrous bands [12, 54, 86].

The improvement in tremors was borderline. There was highly statistically significant improvement in pre-syncope, syncope, and exercise intolerance (Table 2). The improvement in syncope and pre-syncope is presumably due to elimination of mechanical deformative stress of the upper spinal cord or brainstem [35, 42, 43, 69]. Modest improvement—though not statistically significant—was seen in imbalance, vision problems, hands and feet turning cold/blue, and fatigue (Table 3). Symptoms related to hearing, swallowing, and breathing were not improved, nor was leg strength.

The patient satisfaction surveys were generally positive. However, two patients reported dissatisfaction with the surgery and no improvement in quality of life. Given the nature of the survey, the reason for dissatisfaction was not apparent, though the large number of comorbid conditions may have been responsible in part for ongoing disability.

### Behavioral symptoms

There was a surprising pre-operative incidence of depression (17/20) and anxiety (18/20). Highly statistically significant improvement in anxiety and depression followed stabilization. These findings suggest a salutary effect upon the brainstem with respect to the upward transmission to hypothalamus, amyygdala, medial forebrain, temporal, and limbic lobes. Physiological stressors have been shown to activate C1 neurons, and result in global sympatho-excitation [37]. It is reasonable, moreover, to expect that alleviation of the burden of disability, pain, and illness would decrease anxiety and depression.

### The effect of C1-C2 fusion on headache and pain

**Fig. 8:**
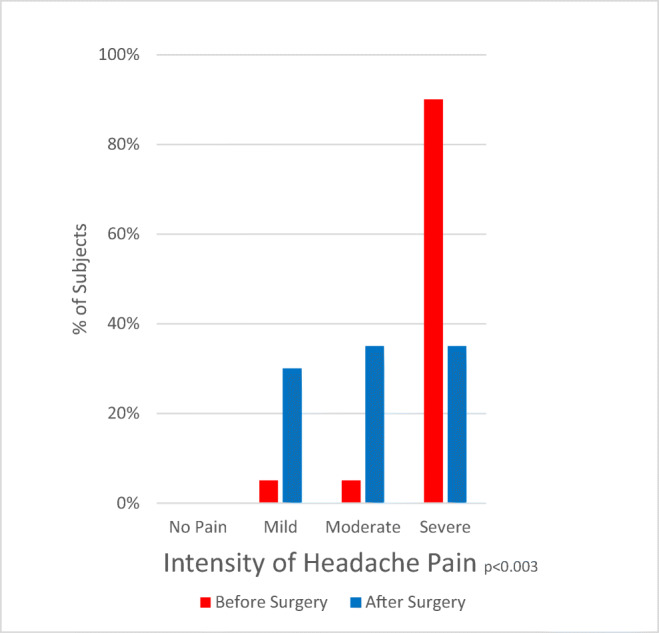
Histograms of intensity of headache show a statistically significant improvement in pain intensity (*P* = 0.03)

**Fig. 9:**
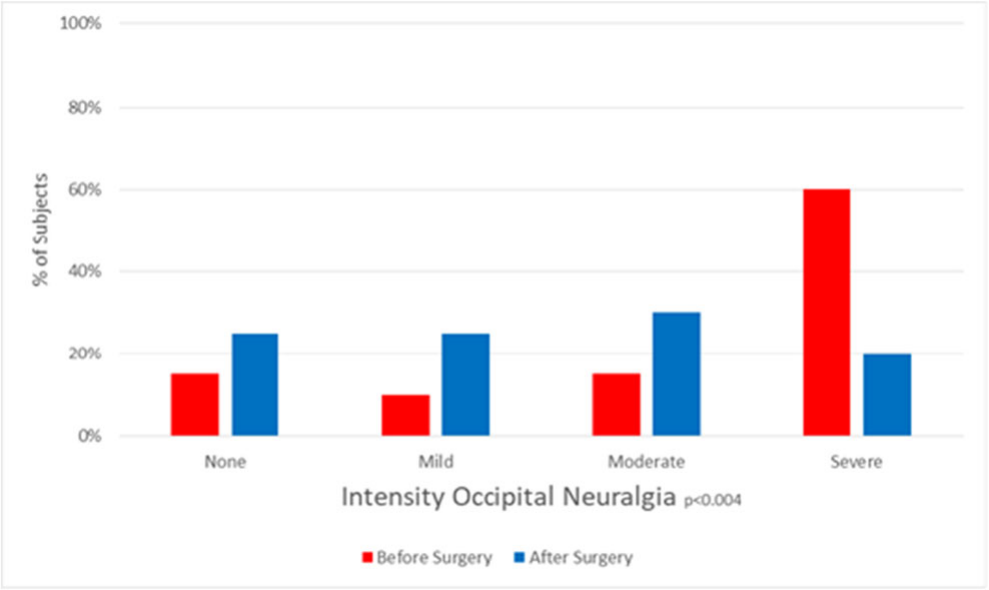
Histograms of intensity of pre-operative and post-operative occipital neuralgia show a statistically significant improvement of neuralgia intensity (*P* < 0.01)

**Fig. 10:**
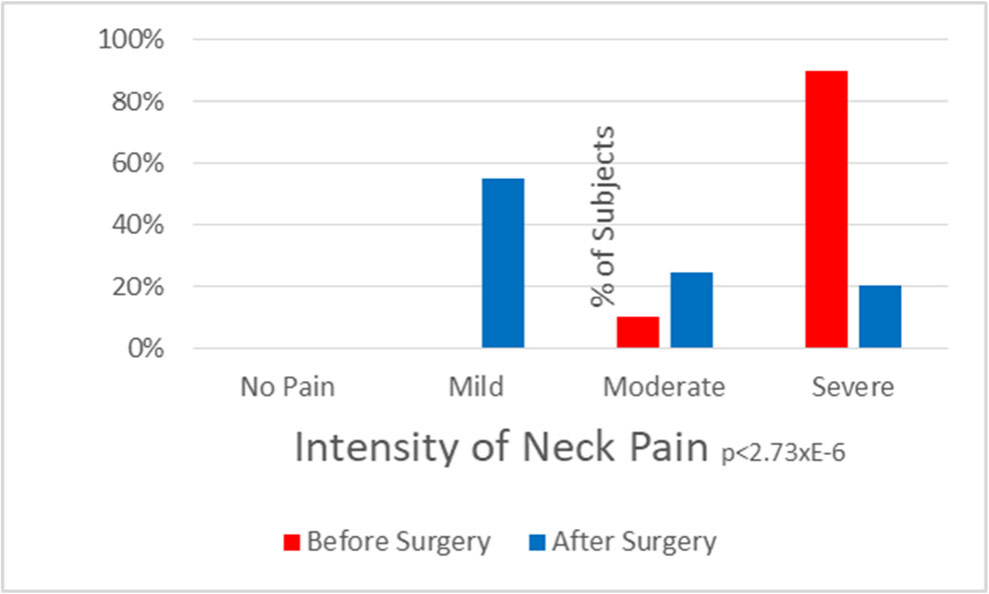
Histograms of pre-operative and post-operative intensity of neck pain show a statistically significant improvement in neck pain intensity post-operatively (*P* < 0.0001)

The improvement of headache (Fig. 8) paralleled the improvement of occipital neuralgia (Fig. 9), suggesting that a significant component of headache results from the greater occipital nerves. Unlike all other cervical roots, the C2 roots exit between the laminae and are more subject to the chronic trauma inherent in highly mobile segments. The improvement in neck pain (Fig. 10) presumably is the logical consequence of stabilization of the unstable motion segment. The less impressive improvement in global pain evident in the global visual analog scale and the Global Impression of Change reflects the significant amount of pain these subjects with EDS were experiencing elsewhere throughout the body.

### Complications of C1-C2 fixation and fusion

Though there were no apparent peri-operative complications, it was necessary to revise the fusions in 2 subjects (10%) over 12 months after surgery. In one case, the necessary revision was due to the patient falling. Prior to the fall, the allograft exhibited early bone growth on CT, the appearance of which was lost after the fall. This occurrence draws attention to the slow mineralization and remodeling of the allograft fusion, which typically requires at least 12 months in this patient population. In a second subject, bone fusion between C1 and C2 was demonstrated on CT at the 6-month post-operative visit, but a broken screw at C1 was noted on CT at 12 months, suggesting that even in the presence of a demonstrably successful fusion, the mechanical stresses upon the C1 level are substantial and capable of causing metal fatigue and fracture, underscoring the potential of increased magnitude of deformative stress at the cranio-cervical junction in this population.

Fusion failure in this patient population is probably higher due to osteopenia and small bone anatomy, and consistent with the higher rate of failed posterior fusion in patients with rotational instability [13].

Larger studies of C1-C2 fusion have described suboccipital numbness (16.8 to 50%) which usually subsides over several months: failed fusion (0.2%); dural tears (0.3%); screw breakout at the atlanto-axial complex (0.4%); screw fracture (0.8%); infection (0.5%); and suboccipital neuralgia (0.1%) [94].

The Goel-Harms technique is associated with a lesser morbidity than transarticular C1-C2 fixation technique. In the latter, Madawi reported screw malposition in 15/61, failed fusion 8/61, screw breakage in 5/61, and cranial nerve injury in 1/61 [60]. The risk of compromise of the vertebral artery is 1.2 to 8.2% per subject undergoing pedicle screw fixation, with a risk of neurological deficit 0.2 to 3.7%, and death 0.1% [22, 60, 73, 94, 96].

There is an increased risk of vascular injury associated with the connective tissue disorders. Paramore reported a *high riding* C2 transverse foramen in 18% of patients [75]. A high riding vertebral artery was present in 50 to 70% of patients with Down syndrome, rheumatoid arthritis, basilar invagination, Klippel-Feil syndrome, and congenital skeletal anomalies [11]. Madawi found that the pedicle anatomy precluded safe screw placement in 20% of patients [60]. Moreover, the risk of vertebral artery injury and stroke is increased when a dominant left vertebral artery—which occurs in 50% of patients—is associated with a contralateral hypoplastic vertebral artery, which occurs in 1.9 to 11.6% of patients [52].

The authors strongly endorse careful study of thin-slice axial and sagittal computerized tomography scans of the C1-C2 complex, to select screw length and trajectory [11, 17].

### Etiology of AAI

The atlanto-axial junction (AAJ) is the most mobile joint of the body. Held together by ligaments that allow a great degree of freedom of rotation, the AAJ is responsible for 50% of all neck rotation, 5° of lateral tilt, and 10 to 20° of flexion/extension [91]. Weakness of the muscles and ligaments, hormonal changes, infection, immunological problems, and congenital dysmorphism may contribute to the overall mechanical dysfunction at the C1-C2 motion segment. An intrinsic defect in collagen fibers results in ligamentous laxity, such that AAI may be the product of chronic trauma “superimposed on congenitally weakened ligaments” such as in Down and Ehlers-Danlos syndromes [41, 42, 61, 77]. AAI was found in 3 of the 4 subjects with EDS-vascular type [38, 48, 78].

The mechanical properties of the C1-C2 motion segment are primarily determined by transverse and alar ligaments, although finite element analysis suggests importance of the tectorial membrane and atlanto-condylar ligaments [83, 87, 93]. The tectorial membrane and accessory atlanto-axial ligaments contribute to passive restraints of the atlanto-axial junction [99]. Incompetence of the cruciform ligament in restraining anterior subluxation of the C1 ring underlies AAI in rheumatoid arthritis and Down syndrome [21, 24, 25, 68, 89]. However, the subjects in this cohort, as in Fielding’s series of 17 subjects with rotary type I AAI, are characterized by alar ligament incompetence. The alar ligament attaches from the odontoid to the occiput and C1 ring, restraining rotation, and lateral tilting [20].

The AAJ is ill-equipped to handle the required multi-axial movements in the presence of ligamentous laxity or disruption. The alar ligament consists chiefly of inelastic collagen fibers, and can be irreversibly stretched after trauma, potentially leading to chronic occult hypermobility of the cranio-cervical junction. The alar ligaments are often injured in motor vehicle collisions and implicated in whiplash-associated disorders. Moreover, the long slender neck in women with h-EDS is more vulnerable in flexion extension injury [4, 6, 87]. Failure of the alar ligament allows a 30% increased rotation to the opposite side [19].

In the adult, there is normally substantially less than 40° of rotation at the C1-C2 motion segment [62, 74, 101]. At 35° of rotation of C1 upon C2, there is stretching and kinking of the contralateral vertebral artery [80], and between 40 and 45°, both vertebral arteries become fully occluded [67]. Extreme rotation of the cervical spine may cause injury to the vertebral arteries, with resulting cerebellar or brainstem stroke, and death [79]. It should be emphasized that the reference range of C1-C2 angular measurements are not established in children, in whom hypermobility is more prevalent.

Motor delay, headache associated with “connective tissue pathological relaxation” and quadriparesis have been attributed to ligamentous laxity and instability at the atlanto-occipital and atlanto-axial joints [49, 55, 71]. This may in part be explained by the decreased canal diameter and increased risk of spinal cord compression with increased C1-C2 angulation. With C1-C2 rotation, *before* the point of dislocation, 39% of subjects exhibit a spinal canal diameter of 1 cm [65]. A canal diameter less than 14 mm predicts both the development and severity of weakness, and should prompt consideration of surgical intervention [8, 53, 100]. Moreover, the small canal diameter that occurs with rotary subluxation causes obstruction of cerebrospinal fluid flow, precipitating headache and neurological deficits [2, 36, 59, 69, 92].

If the atlanto-axial motion segment becomes unstable and sagittal balance is compromised, compensatory subaxial deformity may occur. Atlanto-axial subluxation may decrease lordosis at the C1-C2 segment, such that the cervical spine compensates with increased lordosis to maintain balance [76, 98].

## Limitations

The study was retrospective and limited to a cohort of 20 surgical subjects. Several candidates were excluded because of other concurrent surgeries. There was no control for placebo effect. Though an independent nurse collected the data, there may have been patient recall bias or a tendency to give positive answers. The length of the questionnaires delayed the data return; several subjects did not complete and return the data, and it was necessary to enlist sequential subjects. Comorbid conditions profoundly impacted the majority of subjects and probably influenced the outcome metrics.

## Conclusion

A syndrome of severe occipital headache, syncope or pre-syncope, vision changes (especially impaired peripheral vision), dysesthesias of the extremities, hyperreflexia, and decreased pinprick sensation should prompt consideration of AAI. The diagnosis of rotary AAI (Fielding type 1) is difficult and requires directed workup, including dynamic imaging. Patient satisfaction is high, and there is significant improvement of neurological symptoms associated with appropriate reduction and C1-C2 stabilization.
